# Group-based constraint-induced movement therapy in the rehabilitation of chronic poststroke patients

**DOI:** 10.1097/MD.0000000000024864

**Published:** 2021-02-26

**Authors:** Fábio R.O. Galvão, Maria C.A. Silvestre, Camila L.A. Gomes, Nayara K.F. Pereira, Viviane T.B. Nóbrega, Wellington S. Lima, Afonson L.M. Gondim, Enio W.A. Cacho, Roberta O. Cacho

**Affiliations:** Occupational Therapy, MD, Federal University of Rio Grande do Norte - Faculty of Health Sciences of Trairí, Av. Rio Branco, 435 – Centro, Santa Cruz - Rio Grande do Norte, Brazil.

**Keywords:** constraint-induced movement therapy, occupational therapy, recovering after stroke, stroke rehabilitation, task performance, upper extremity

## Abstract

**Background:**

This study aimed to describe the effects of a 90-minute group-based constraint-induced movement therapy protocol (CIMT) on upper extremity (UE) rehabilitation in poststroke patients.

**Method:**

The study was a case series clinical study with 6 patients with chronic stroke admitted to the institutional integrated clinic. Ten 90-minute CIMT sessions were administered, based on the principles of the original therapy. On completion of the protocol, participants underwent group care once a week, for 1 hour a day. For comparison purposes, the Wolf Motor Function Test (WMFT), Motor Activity Log (MAL), and Canadian Occupational Performance Measure (COPM) were applied on admission, shortly after completing the protocol, and 3 months after completion. In addition, the MAL and shaping tasks were applied daily.

**Results:**

There was a statistically significant difference only in the MAL Amount of Use Scale applied daily between the 2nd (3.56) and 9th (3.31) and 2nd and 10th days (4.49) (*P* = .004), with a moderate effect size (d’ = 0.46), and in the average value of shaping repetitions between the 1st (16.10) and 2nd (6.00) and 1st and 10th tasks (7.00) (*P* = .014), with a moderate effect size (d’ = 0.35).

**Conclusion:**

The 90-minute CIMT protocol resulted in significant improvements in use of the more affected arm in activities of daily living during the 2-week protocol. Additional research with a larger sample and a control group is needed to confirm its effectiveness.

## 1 Introduction

Constraint-induced movement therapy (CIMT) is an evidence-based technique recommended by the American Heart Association to recover the movement and function^[1]^ of injured upper extremities (UEs) after stroke.^[2]^ It is based on 3 principles: repetitive, intensive task-oriented training for several hours a day, through small activities called shaping; behavioral methods (transfer package) obtained during training and transferred from the clinical environment to the patient's real environment, through functional activities (task practice); and restriction of the non-paretic UE for 90% of waking hours, using a restriction glove.^[3]^ For shaping in particular, the method involves progressively increasing the difficulty of the tasks as patient performance improves by providing positive graduated feedback.^[4,5]^ In the original protocol, therapy is performed 6 hours a day over 2 consecutive weeks.^[6]^

Due to difficulties in implementing the original approach, a systematic review by Fleet et al^[7]^ analyzed 15 modified protocols with different execution times. The authors concluded that modified CIMT (mCIMT) is an effective intervention for UE recovery after chronic stroke.

In addition to mCIMT, another alternative to overcome these difficulties is through a group therapy protocol. The transition from an individual to a collective intervention improves the social support network and achieves the principle of universality, providing a broad basis for the exchange of ideas and emotional support, stimulating the rehabilitation process.^[8]^ The interaction between participants increases self-esteem, improves functional capacity and performance in important activities, and establishes social relationships, helping them overcome their difficulties.^[9]^ In a randomized clinical trial by Doussoulin et al,^[8]^ group-based CIMT participants reported that the format allowed them to interact with individuals with similar disabilities and obtain feedback on their experiences, in addition to providing an opportunity for collaborative work, which influenced their functional independence at home and in the community.

There are no studies that describe only the benefits of a modified 90-minute protocol, except for a comparative study between this format and 3-hour CIMT.^[10]^ In addition, use of this therapy in a group setting for poststroke patients is scarce. It is believed that a shorter protocol associated with a group approach will have the same benefits as the original format, but generate better treatment adherence and motivation as well as lower costs. Thus, the aim of this study was to describe the effects and feasibility of a 90-minute CIMT protocol, in a day camp setting, in UE rehabilitation of poststroke patients.

## 2 Methods

This was a clinical case series with 6 participants in a single group, treated at the Integrated Clinic of the Faculty of Health Sciences of Trairi (FACISA, UFRN), in Santa Cruz, Rio Grande do Norte state, Brazil. Convenience sampling was used.

The study was carried out from January to May 2019 and approved by the Human Research Ethics Committee of the Faculty of Health Sciences of Trairi (FACISA - UFRN) on November 12, 2018 under protocol number CAAE 3.015.121. It was retrospectively registered with the Brazilian Registry of Clinical Trials (RBR-2q98vj) and approved on July 4, 2019. All participants provided written informed consent, in accordance with the principles of the Declaration of Helsinki, and the study was in line with Standard Protocol Items: Recommendations for Interventional Trials guidelines.

### 2.1 Participants

Inclusion criteria were clinical diagnosis of ischemic or hemorrhagic stroke, time since stroke > 6 months, age over 18 years, unilateral sequelae according to the functional criteria of the original protocol, as follows: active movement of at least 45° of shoulder flexion and abduction, 20° of elbow extension, 10° of wrist extension, 10° of thumb abduction/extension and at least 10° of extension in 2 other fingers, based on goniometric assessment. Individuals who had participated in another upper limb rehabilitation study or exhibited some form of cognitive impairment (Mini-Mental <13 for illiterates, <18 for low or medium schooling level, and <26 for high levels) were excluded.^[11]^

**Figure 1:**
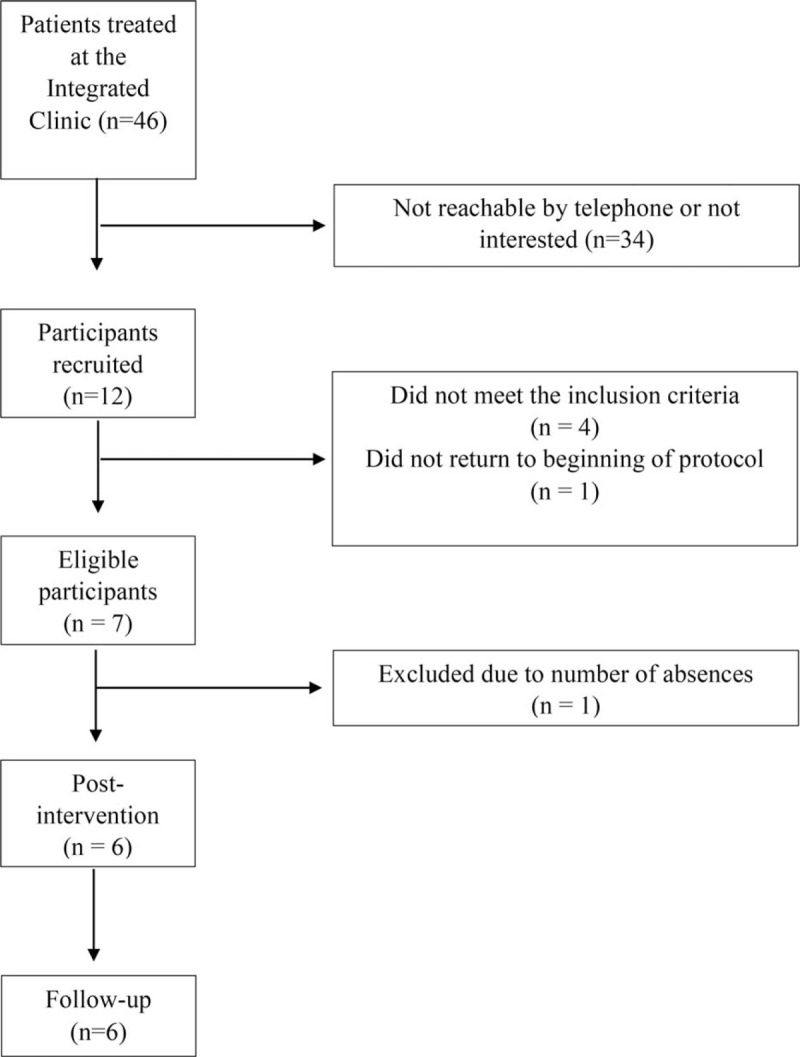
Flowchart of research participants.

Following telephone contact, initial assessment and determination of eligibility based on inclusion and exclusion criteria and excessive absenteeism (3 or more missed sessions), of the 46 individuals registered at the clinic, only 6 were selected (Fig. 1).

### 2.2 Procedures

The following clinical instruments were applied by an independent therapist on admission, shortly after completing the protocol, and 3 months after completion:

#### 2.2.1 Wolf Motor Function Test (WMFT)

WMFT developed to assess the effects of CIMT in patients with stroke. Upper limb mobility is evaluated in 17 tasks, considering strength, functional capacity, and execution time, progressing from simple proximal to complex distal joint movements, in a maximum time of 120 seconds per task. The total score ranges from 0 to 85, whereby the higher the score, the greater the functionality. The instrument was translated and adapted for Brazilian Portuguese and showed reliability for assessing paretic UE functionality in adults with hemiparesis.^[12]^ The WMFT scores were defined as the primary outcome in this study.

#### 2.2.2 Motor Activity Log (MAL)

MAL assesses the amount of use (AOU) and quality of movement (QOM) in 30 activities of daily living, which individuals rate according to their own reality. Scoring is on a scale of 0 to 5, whereby the higher average score, the better the movement.^[13]^ This instrument was adapted and validated for Brazil for use in patients with chronic stroke, and found to be a satisfactory measure of UE performance in individuals with mild and moderate motor impairments.^[14]^

#### 2.2.3 Canadian Occupational Performance Measure (COPM)

COPM is a semi-structured interview designed to capture patients’ self-perception of occupational performance in activities of daily living, taking their health conditions into account.^[15]^ Patients assess the level of importance of activities related to self-care, productivity and leisure, as well as satisfaction with their performance in these tasks.^[16]^ First, they are asked to rate the activities from 1 (not important at all) to 10 (extremely important) in terms of importance. Next, the evaluator and patient identify the 5 most important problems perceived in daily activities, rating performance from 1 (not able to do it at all) to 10 (able to do it extremely well) and satisfaction, also from 1 (not satisfied at all) to 10 (extremely satisfied). Average performance and satisfaction scores are calculated by summing the individual activity scores and dividing by the number of activities.^[17]^ In addition to its use as a comparative clinical instrument, the COPM was also applied to analyze the difficulties of each participant and select the shaping tasks accordingly.

### 2.3 Intervention

In a group format and summer camp approach, the participants underwent ten 90-minute morning sessions a day from Monday to Friday, over 2 consecutive weeks, in line with CIMT methodology. The activities were supervised by 2 occupational therapists, a physiotherapist and 10 undergraduate physiotherapy students, who received prior training in application of the protocol.

At the start of the protocol, each participant was given a containment glove made of fabric to be used during the 2-week protocol in both the therapy sessions and home-based activities, and explained the form and importance of its use. Participants were randomly divided into 3 stations, each with different shaping tasks. These were repeated throughout the protocol, in rotation, so that all participants performed the same tasks on the same day, with task practice carried out between shaping tasks. At the end of the first day, participants signed a behavioral contract whereby they agreed to perform some activities at home and use the glove for 90% of waking hours. This contract is a formal agreement between the patient and therapist and forms part of the CIMT protocol.^[3]^ As part of the contract, 3 tasks were selected for subjects to practice at home.

From the second to the last day of the protocol, participants were evaluated using the MAL-AOU. The 3 activities they were unable to do or performed with some degree of difficulty were assigned as home-based tasks. For those that they performed well, the level of difficulty was increased or new tasks were prescribed.

A total of 30 shaping and ten practice tasks were performed, selected based on the difficulties identified in COPM assessment. Each shaping task was executed ten times, at 45 seconds each, timed by the therapist. Patients were given feedback on the repetitions, which were quantified on a scale from 0 to 5, according to the QOM scale. As patient performance improved, the difficulty of each task was increased through graduated feedback. For comparison purposes, 1 shaping per day (related to fine motor skills) was chosen, totaling ten shaping tasks. These were performed at random throughout the program and not graded for difficulty (see Table, Supplemental Digital Content 1, which demonstrates the 30 shaping tasks selected). Both the shaping and practice tasks were carried out collectively and consisted of recreational activities, preparing meals or snacks, simulating buying groceries at the supermarket and walking in the community. These activities were repeated over the 2-week period.

On completion of the protocol, participants were submitted to group care once a week for 1 hour a day, over 5 months, consisting of conventional exercises that targeted mobility and balance.

Risk during the intervention was considered minimal by the researchers and related to feeling uncomfortable performing activities in front of the group and fatigue during sessions, as reported by the patients. Should this occur, the therapist interrupts the session and reschedules it to a more convenient time for the participant. The team of professionals were available to assist participants in the activities during the protocol and could interrupt the session whenever they deemed it necessary.

### 2.4 Statistical analyses

Statistical analyses were carried out with *BioEstat* 5.3 software. Significance was set at *P* < .05. Data normality was assessed by the Shapiro–Wilk and nonparametric tests. The sample was characterized using descriptive analysis of the variables, with values expressed as median, quartiles, and percentage. The difference between the results of clinical instruments (MAL, WOLF, and COPM) before and after intervention and at follow-up were assessed using the Friedman test, since the data were nonparamedic.

The Friedman test was also used to analyze the daily difference between MAL-AOU applied at the beginning of each session, and statistically significant differences between shaping tasks performed over the 10 days.

The effect size was determined based on 3 categories, namely, 0.2 = small, 0.5 = moderate, and 0.8 = large.^[18]^

## 3. Results

3

The characteristics of the sample are presented in Table 1, which demonstrates that most participants were elderly, male, with a low schooling level and ischemic stroke in the chronic phase. Half of the participants presented 1 side as the most compromised, with no significant disabilities according to the modified Rankin Scale^[19]^ and mild sensory motor impairment, in line with the Fugl-Meyer Upper Extremity Scale.^[20]^ No participants obtained scores indicating cognitive deficit based on the Mini-Mental State Examination (MMSE).

**Table 1:** Sample characterization.

Participantn = 6	Age	Gender	Schooling level	Stroke Type	Paretic Hand	Time since stroke (years)	Rankin Scale	FMA-UL	MMSE
1	62	M	PEC	I	R	13	1	57	19
2	50	M	IL	H	R	3	2	35	20
3	73	F	PEI	I	R	4	1	52	20
4	78	F	IL	H	L	2	2	23	22
5	76	M	IL	I	L	1	1	53	25
6	64	M	HSC	I	L	0.6	1	53	25
Median	68.50	-	-	-	-	2.50	1.00	52.50	21
(1Q/3Q)	59/76.50	**-**	**-**	**-**	**-**	0.90/6.25	1.00/2.00	32.00/54.00	19.75/25.00
Values (%)	-	M = 57.1F = 28.6	I = 42.9PEI = 14.3PEC = 14.3HSG = 14.3	H = 14.3I = 71.4	R = 50L = 50	-	-		-

Comparative analysis between variables of the MAL (quantity and quality), WMFT (time, strength and qualitative scale), and COPM (performance and satisfaction) is shown in Table 2, with no statistical significance between the clinical scales.

**Table 2:** Comparative statistical analysis of the clinical instruments.

Clinical Instruments	Admission Mediana (1Q/3Q)	PostTreatment Mediana (1Q/3Q)	Follow-Up Mediana (1Q/3Q)	Effect Size	*P* value
WMFT - Time	3.79 (2.51/9.04)	3.90 (3.23/4.61)	3.68 (2.69/4.77)	0.11	.513
WMFT – Strength	30.66 (26.57/47.28)	33.41 (26.83/39.30)	32.08 (20.74/64.45)	0.00	1.000
WOLF - Qualitative	4.76 (3.44/4.95)	4.73 (4.15/4.95)	4.35 (3.86/5.00)	0.10	.538
MAL – Quantity	3.49 (2.44/3.99)	3.53 (2.97/4.34)	3.31 (2.88/3.65)	0.11	.513
MAL - Quality	3.16 (1.70/3.86)	3.39 (2.52/4.26)	3.17 (1.25/3.81)	0.19	.311
COPM - Performance	7.60 (6.75/8.57)	7.85 (7.05/8.62)	8.20 (5.95/9.25)	0.24	.229
COPM – Satisfaction	8.00 (7.90/8.45)	8.00 (7.90/8.45)	8.60 (7.15/9.85)	0.34	.129

Table 3 depicts the statistical analysis of the MAL-AOU. According to the table, there was a significant difference between the 2nd (3.56) and 9th (3.31) and 2nd and 10th days (4.49) (*P* = .004), with a moderate effect size (d’ = 0.46).

**Table 3:** Analysis of the MAL Amount of Use Scale, applied daily in median and quartiles.

Day	Day 2	Day 3	Day 4	Day 5	Day 6	Day 7	Day 8	Day 9	Day 10			d’	*P* value
MAL-AOU	3.56^ab^ (2.23/3.84)	3,85 (2.63/4.18)	3.89 (2.58/3.39)	3.61 (2.66/4.65)	3.77 (2.98/4.14)	3.67 (3.02/4.32)	4.31 (3.46/4.36)	3.31^a^ (3.60/4.53)	4.49^b^ (3.52/5.56)			0.46	.004

Statistical analysis of the QOM, task progression, and average repetition of shaping tasks is presented in Table 4, with a significant difference for the repetition means between tasks on the 1st (16.10) and 2nd (6.00) and 1st and 10th days (7.00) (*P* = .014), with a moderate effect size (d’ = 0.35).

**Table 4:** Analysis of the quality of movement, task progression, and average repetition of shaping tasks.

Median (1Q/3Q)	Day 1	Day 2	Day 3	Day 4	Day 5	Day 6	Day 7	Day 8	Day 9	Day 10	Effect Size	*P* value
RA	16.10^a,b^ (11.12/ 19.72)	6.00^a^ (4.67/ 10.00)	7.95 (3.82/ 9.27)	7.45 (4.12/ 11.40)	7.75 (5.55/ 8.80)	9.35 (5.55/ 8.80)	12.35 (5.85/ 17.62)	5.05 (2.85/ 11.32)	10.35 (6.45/ 12.12)	7.00^b^ (4.72/ 9.12)	0.35	.002
MQ	4,00 (3.75/ 4,62)	4.00 (4.00/ 4.00)	4.00 (2.25/ 4.10)	4.25 (2.36/ 5.00)	4.50 (3.00/ 4.92)	4.00 (3.00/ 4.45)	3.95 (2.62/ 4.92)	4.00 (3.97/ 4.70)	4.50 (4.00/ 4.92)	4.00 (4.00/ 4.35)	0.14	.535

## 4 Discussion

In the present study, CIMT was not effective in the rehabilitation of poststroke patients.

The professionals responsible for the CIMT program observed that not only were the participants self-motivated (intrinsic motivation), but they encouraged others in the group to successfully complete each task, creating a relaxed and enjoyable atmosphere. Healthy competition between groups was also observed, with participants competing to see who performed the tasks best. Increased bonding and the exchange of experiences also contributed to overcoming difficulties and improving the potential of the therapy. Participants also showed great patience in using their more affected arm, which, depending on the difficulty of the tasks, meant they often completed them satisfactorily, even when this required developing new strategies for each task. Finally, when practicing the tasks in a group, patients were seen helping others who had difficulty completing the tasks independently.

In the present study, no significant difference was found in the MAL, WMFT and COPM clinical scales between the pretreatment, posttreatment, and follow-up periods, or for the daily MAL, except the daily MAL-AOU and mean values for repeated shaping tasks. This suggests that the 90-minute protocol may not be sufficient to rehabilitate these patients due to the short clinical treatment time, or that they had already achieved maximum functionality.

In a comparative study, Souza et al^[10]^ reported that both the 90-minute and 3-hour protocols were feasible for rehabilitation purposes. To date, there are no investigations that describe only the effects of a 90-minute protocol. Studies have shown that shorter CIMT models (1–2 hours) proved to be as effective as the original protocol.^[21–23]^

With respect to injury time, Wahl and Schwab^[24]^ observed spontaneous recovery in poststroke patients in the first weeks or months after the injury, with the greatest recovery occurring in the first 3 to 6 months. Participants in the present study had a median injury time of 3 years and most had taken part in previous CIMT experiments. Thus, we hypothesize that these subjects may have achieved maximum functional recovery. According to Baldwin, et al,^[25]^ CIMT is best applied to patients in the early stages of recovery; however, some studies have also reported functional gains in chronic patients.^[26–28]^

In regard to the daily MAL-AOU, there was a statistically significant difference between the 2nd and 9th and 2nd and 10th (4.49) days of the protocol, with average values of 3.56 and 3.31, respectively. This indicates an improvement with a small oscillation followed by an increase in use of the more affected arm for activities performed at home between these days. This corroborates the learned nonuse phenomenon reported by Taub et al.^[6]^ According to Page et al,^[29]^ as participants progressed in CIMT, they realized they were capable of using the more affected arm in their daily activities more frequently than they previously thought. At least 3 of our participants showed improvement in the activity of removing a piece of clothing from a drawer, 3 for cleaning a kitchen counter or other surface and 3 when drying their hands.

The format used for the shaping tasks may have influenced the results, since there was only a significant difference in the average values between tasks on the 1st and 2nd and 1st and 10th days, with values of 16.10 (day 1), 6.00 (day 2) and 7.00 (day 10). As such, we concluded that the task performed on the first day was easier than the others, based on the average values recorded. It should be noted that the tasks were not performed according to the degree of difficulty over the 2-week period.

According to Abdullahi,^[30]^ the number of repetitions in shaping tasks can improve both the motor function and use of UEs in everyday activities. Kolobe et al^[31]^ reported that the frequency, intensity and duration of the intervention may influence treatment effectiveness, depending on the protocol used.

### 4.1 Limitations

Study limitations were the lack of standardization in grading the difficulty of shaping tasks, small sample size and absence of a control group not submitted to mCIMT, which may have influenced the results. In light of the fact that the protocol required participants to attend sessions every day of the week over 2 consecutive weeks, many were unable to adhere to the treatment. Additionally, some lived in neighboring cities or rural areas and did not have their own means of transport, making it difficult for them to take part. The individuals studied may also have achieved their maximum functional gain because of the time since stroke and their participation in previous CIMT protocols and other therapies.

## 5 Conclusion

Although some findings were not statistically significant, the 90-minute CMIT in a day camp model seems to be a promising rehabilitation model. Use of the more affected arm in activities of daily living increased during the 2-week protocol. Moreover, the group interacted well and the atmosphere was relaxed and enjoyable, which helped participants overcome their difficulties and optimized the potential of the therapy.

As such, it is recommended that this study be replicated with a larger sample, preferably compared to a control group, taking into account the grading of shaping tasks, injury time, and participation in other therapies or previous CIMT protocols.

## Author contributions

**Conceptualization:** Fábio R.O. Galvão, Maria C.A. Silvestre, Roberta O. Cacho.

**Data curation:** Fábio R.O. Galvão.

**Formal analysis:** Fábio R.O. Galvão, Wellington S. Lima Junior, Enio W.A. Cacho.

**Investigation:** Fábio R.O. Galvão, Camila L.A. Gomes, Nayara K.F. Pereira, Viviane T.B. Nóbrega, Afonson L.M. Gondim.

**Methodology:** Fábio R.O. Galvão, Maria C.A. Silvestre, Roberta O. Cacho.

**Project administration:** Fábio R.O. Galvão.

**Resources:** Fábio R.O. Galvão.

**Supervision:** Fábio R.O. Galvão, Enio W.A. Cacho, Roberta O. Cacho.

**Validation:** Fábio R.O. Galvão.

**Visualization:** Fábio R.O. Galvão.

**Writing – original draft:** Fábio R.O. Galvão.

**Writing – review & editing:** Roberta O. Cacho.

## Correction

When this article originally published, Dr. Enio W.A. Cacho's name appeared incorrectly as Ênio W.A. Cacho. This has since been fixed.

In table 4, the p value in the first line was misprinted as 0.026 and has been corrected to 0.002.

## Supplementary Material

Supplemental Digital Content
