# Supplementary material for: Group-based constraint-induced movement therapy in the rehabilitation of chronic poststroke patients
Source: Medicine (Baltimore). 2021 Feb 26;100(8):e24864. doi: 10.1097/MD.0000000000024864 (PMC7909176; doi:10.1097/MD.0000000000024864)
Supplement: Supplemental Digital Content [file medi-100-e24864-s001.docx]

|  |  |  |  |
| --- | --- | --- | --- |
| Tasks Description | Movements | Parameters | Progression |
| Task 1: Rings in a cup  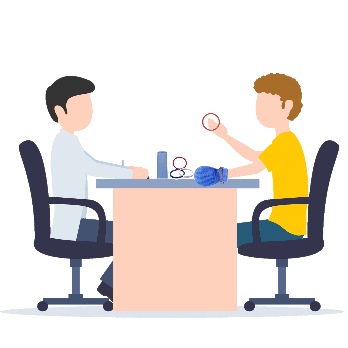 | Reach and grasp, wrist and elbow extension. | Number of rings (initial target) reached, removed, transported and placed around the cup (final target) in 40 seconds. | Increase the distance between targets;  Increase the base where the cup was positioned;  Place the ring next to the patient’s most affected. |
| Task 2: Fork  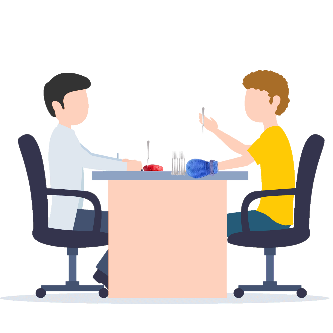 | Reach and grasp, forearm supination, pronation, adduction and abduction. | Number of forks (initial target) reached, removed and transferred to the modelling clay (final target) in 40 seconds. | Increase the distance between targets;  Raise the height of one of the targets. |
| Task 3: Stick  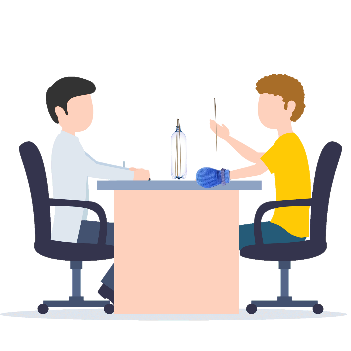 | Pinch movement, elbow flexion and extension, forearm supination and pronation, shoulder flexion and extension. | Number of sticks reached and removed from a bottle (initial target) and transferred to another bottle (final target) in 40 seconds. | Increase the distance between targets;  Raise the height of one of the targets.  Place the bottle next to the patient’s most affected hand. |
| Task 4: Card  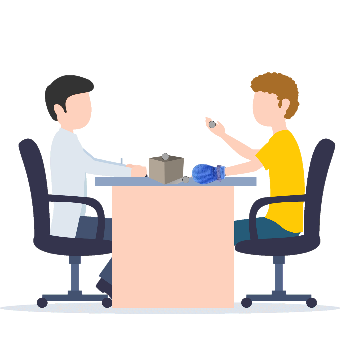 | Pinch movement, forearm pronation, shoulder/elbow flexion and extension. | Number of cards (initial target) reached, removed and placed in a box (final target) in 40 seconds. | Increase the distance between targets;  Raise the height of one of the targets. |
| Task 5: Rubber bands  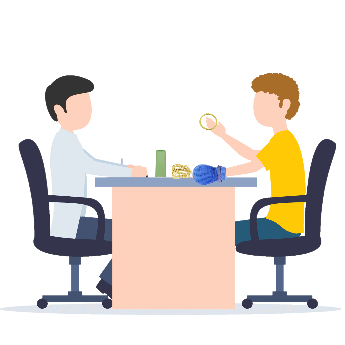 | Reach and grasp, finger extensions, wrist flexion and extension, elbow extension. | Number of rubber bands (initial target) the patient was able to place around a cup (final target) in 90 seconds. | Increase the distance between targets;  Increase the diameter of the cup;  Increase the distance between targets; |
| Task 6: *Hashi* (Japanese cutlery)  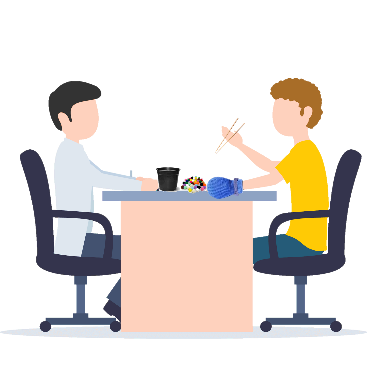 | Pinch movement, elbow / shoulder adduction, abduction, flexion and extension. | Number of little balls transferred from a plate (initial target) to a cup (final target) in 40 seconds using a “hashi”. | Increase the distance between targets;  Raise the height of one of the targets. |
| Task 7: Beans  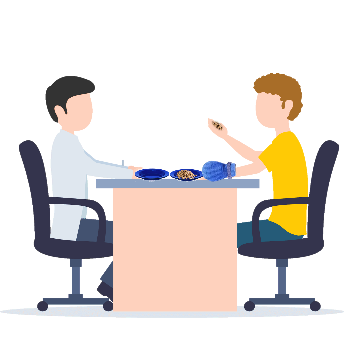 | Reach and grasp, forearm pronation and supination, elbow abduction and adduction, elbow / shoulder flexion and extension. | Number of beans transferred from a plate (initial target) to another plate (final target) in 40 seconds. | Increase the distance between targets;  Raise the height of one of the targets. |
| Task 8: Button up a shirt  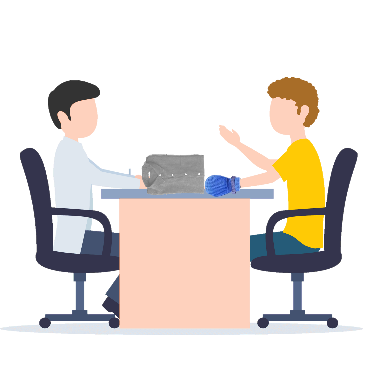 | Pinch movement and wrist extension. | Number of buttons that were buttoned in 45 seconds. | Raise the height of the shirt or position it further from the participant. |
| Task 9: Washing line  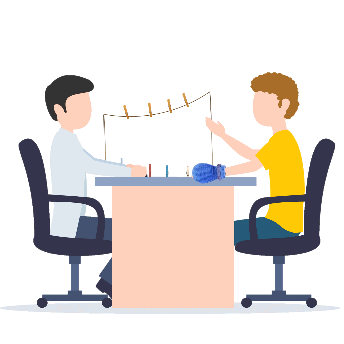 | Pinch movement, elbow flexion and shoulder extension. | Number of clothes pegs taken from the washing line (initial target) and placed on a plate (final target) in 40 seconds. | Increase the height of the washing line;  Move the washing line away from the participant;  Place the washing line next to the patient’s most affected hand. |
| Task 10: Marble (toy)  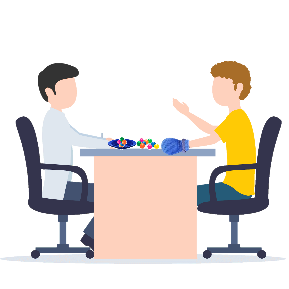 | Pinch movement, forearm adduction / abduction. | Number of marbles (initial target) grasped, transported and placed in the ice mould in 40 seconds using tweezers. | Increase the distance between targets;  Raise the height of one of the targets. |

Supplemental Digital Content 1 Shaping task
